# Exploring Microbial Resource of Different Rhizocompartments of Dominant Plants Along the Salinity Gradient Around the Hypersaline Lake Ejinur

**DOI:** 10.3389/fmicb.2021.698479

**Published:** 2021-07-12

**Authors:** Junqing Luo, Zhechao Zhang, Yazhou Hou, Fengwei Diao, Baihui Hao, Zhihua Bao, Lixin Wang, Wei Guo

**Affiliations:** Inner Mongolia Key Laboratory of Environmental Pollution Control and Waste Resource Recycle, Ministry of Education Collaborative Innovation Center for Grassland Ecological Security, Ministry of Education Key Laboratory of Ecology and Resource Use of the Mongolian Plateau, School of Ecology and Environment, Inner Mongolia University, Hohhot, China

**Keywords:** extreme environments, salt stress, soil properties, rhizosphere soil microorganisms, root endophytic microorganisms

## Abstract

Lake littoral zones can also be regarded as another extremely hypersaline environment due to hypersaline properties of salt lakes. In this study, high-throughput sequencing technique was used to analyze bacteria and fungi from different rhizocompartments (rhizosphere and endosphere) of four dominant plants along the salinity gradient in the littoral zones of Ejinur Salt Lake. The study found that microbial *α*-diversity did not increase with the decrease of salinity, indicating that salinity was not the main factor on the effect of microbial diversity. Distance-based redundancy analysis and regression analysis were used to further reveal the relationship between microorganisms from different rhizocompartments and plant species and soil physicochemical properties. Bacteria and fungi in the rhizosphere and endosphere were the most significantly affected by SO_4_^2–^, SOC, HCO_3_^–^, and SOC, respectively. Correlation network analysis revealed the potential role of microorganisms in different root compartments on the regulation of salt stress through synergistic and antagonistic interactions. LEfSe analysis further indicated that dominant microbial taxa in different rhizocompartments had a positive response to plants, such as *Marinobacter*, *Palleronia*, *Arthrobacter*, and *Penicillium*. This study was of great significance and practical value for understanding salt environments around salt lakes to excavate the potential microbial resources.

## Introduction

Salt lakes belong to extremely hypersaline environments and they are widely distributed all over the world. The salt lakes account for about half of the total inland aquatic ecosystems (Yang et al., 2016). For China, salt lakes are generally distributed in northwest China, such as Tibet, Qinghai, Xinjiang, and Inner Mongolia (Zheng et al., 1993). Lakes in these areas provide water resources and play an important role in the fragile environment (Zhang et al., 2020). In addition, although salt inhibits the diversity of microorganisms, certain microorganisms living in salt lakes still have high activity and involve many geochemical cycles necessary for life, such as carbon, nitrogen, and sulfur (Sorokin et al., 2014). Therefore, salt lakes are often considered as one of the best choices for studying the relationship between microbial diversity and environmental factors in extremely hypersaline environments (Kalwasinska et al., 2019). Importantly, due to hypersaline properties of salt lakes, the microbial communities inhabited in lake littoral zone soils may present particularity. The lake littoral zone is a functional transition zone connecting lake aquatic ecosystem and terrestrial ecosystem (Kou et al., 2020). Thus, the lake littoral zones of salt lakes are similarly regarded as an extremely high salt environment.

There is well-known basic work on the genetic diversity and structure of microbial communities in salt lakes, in which halophilic microorganisms (including bacteria and archaea) isolated and identified from the salt lakes and their sediments can be regarded as an important part of biological community (Guven et al., 2018; Selivanova et al., 2018). At present, most of the sampling points in these studies are from estuaries, salt ponds, and sediments of salt lakes (Wu et al., 2006; Boujelben et al., 2012; Liu et al., 2015). However, few researchers have attended to the bacterial and fungal diversity in the littoral zone soils of salt lakes. Salinity is the primary environmental determinant for microbial communities in different saline ecosystems, salt lakes, and other saline soils (Oren, 2008; Hrynkiewicz et al., 2015; Zhong et al., 2016). The salinity increase had negative impacts on soil microbial activity (Chen et al., 2017). However, it is unknown how soil microbial communities change along the salinity gradients in the littoral zones of salt lakes, because there are different dominant plant species along the salinity gradient in the littoral zones of salt lakes (Williams, 1991; Apaydin et al., 2009). Therefore, it is important to ascertain the dominant factors for the diversity and structure of the microbial community under different salinities in the littoral zone of the salt lakes.

Several studies have shown that soil microorganisms exert important effects on maintaining soil health and helping plants resist adverse environments (Wang et al., 2016; Jiang et al., 2019). Beneficial microorganisms can promote plant growth under salt stress. Rhizosphere bacteria from *Salicornia strobilacea* has been shown to settle stably on the root surface and promote the growth of its host plants (Yamamoto et al., 2020). Fan et al. (2016) found that salt-tolerant bacteria isolated from the rhizosphere of *Suaeda salsa* growing on saline soil can be used to alleviate the salt stress of crops. Many studies have revealed that certain bacteria play beneficial effects in plants and regulate a variety of direct or indirect mechanisms by the ways of rhizosphere colonization at the high-salt conditions (Paul and Lade, 2014; Mallick et al., 2018). Ali et al. (2014) found that endophytic bacteria containing 1-Aminocyclopropane-1-carboxylate deaminase (ACC deaminase) can promote plant growth and development under salt stress, but some endophytic fungi from saline–alkaline soils usually stimulate plant growth and give plants tolerance to biotic or abiotic stresses (Khan et al., 2011; Pan et al., 2018). Recent works have successfully used the cooperative relationship among plants and rhizospheric and endophytic bacteria to effectively remediate soil (Fatima et al., 2015; Hussain et al., 2018). Therefore, it is crucial to understand the microbial composition from different rhizocompartments of the plants in hypersaline soil environments, particularly around salt lakes. Exploring the mode of different rhizocompartments of microorganisms of dominant plants in the littoral zones of salt lakes is important to understand how microorganisms help plants resist saline stress and maintain the stability of different saline environments.

In recent years, high-throughput sequencing has been widely used to determine the diversity and structure of microbial communities, understanding the interaction and adaptability of microbes in the environment (Valenzuela-Encinas et al., 2008; Han et al., 2017). In this study, the salt lakes areas of Inner Mongolia Plateau have naturally formed a good soil salinity gradient, and it has become the better experimental platform for studying the microbial diversity in different rhizocompartments of various plants (Du et al., 2007). Through the investigation, dominant plants were discovered to have obvious regional divisions along the salt gradient changes, and the salinization degree of the littoral zone soil environments decreased gradually from the edge of the lake to the periphery. Therefore, we investigated the microbial communities of rhizosphere soil samples and root endophytic samples of four different plants under salinity gradient around Ejinur Salt Lake. Our aim was to (1) study the difference and relationship of rhizosphere soil and endophytic microbial community structure under salinity gradient; (2) explore the dominant microorganisms that can resist salt stress in plants in different soil environments of the littoral zones of salt lakes along the salinity gradient; and (3) reveal the dominant factors affecting microorganisms in different rhizocompartments. This study provided new insights for in-depth understanding of the roles of microorganisms, which was beneficial to excavate the potential microbial resources resistant to saline–alkali stress in the littoral zones of salt lakes.

## Materials and Methods

### Study Sites and Soil Preparation

The Ejinur Salt Lake is located in the north of Dabusu basin in Inner Mongolia Plateau, China (116°32′44′′E, 45°14′34′′N), which is characterized by a typical temperate semi-arid continental climate. The average annual precipitation is 255 mm, and the soil types primarily belong to the saline–alkali soil of chloride type. Rhizosphere soils were sampled along the salinity gradient in the littoral zones of Ejinur Salt Lake in September 2019. Four sampling points [rhizosphere soil sample points were marked as *S. europaea* rhizosphere soils (GA), *S. salsa* rhizosphere soils (GB), *Phragmites communis* rhizosphere soils (GC), and *Achnatherum splendens* rhizosphere soils (GD)] were set up along the salinity gradient, and the plant species changed with the distance from the lake shore. Each sample point was composed of three sub-sample points (Rath et al., 2019) (a single sample point uses the four corners of a 10 m × 10 m quadrilateral and a center point to collect plants sample, and the three points on the diagonal are collected rhizosphere soil samples) at four sample points of the salinity gradient. We excavated whole plants including the surrounding soil in blocks. The collected soil and plant samples were stored in an incubator at 4°C and transported them to the laboratory.

The soil close to the roots (0–3 mm from the roots’ surface) was defined as the rhizosphere soil (Zhang and Kong, 2014; Tian and Zhang, 2017). The rhizosphere soil samples in the root zone were gently separated from the roots according to the method described by Hrynkiewicz et al. (2010). Subsequently, the separated rhizosphere soil samples were air-dried and sieved through a 2-mm sieve and used to determine physical and chemical indicators. In the aseptic operating conditions, the plant roots with firmly attached soil were put into a centrifuge tube containing 50 ml of sterile water and shaken using a shaker (4°C) (Simmons et al., 2018; Wang et al., 2020). After the plant roots were removed from the centrifuge tube, the centrifuge tube containing soils was centrifuged (4°C, 8,000 rpm) for 10 min (Edwards et al., 2015). After the supernatants were removed, the remaining sediments were used as rhizosphere soils for microbial determination. The plant roots were soaked in 70% ethanol for 2 min and 2.5% NaClO for 5 min and then transferred to 70% sterile ethanol and soaked for 30 s. Finally, the plant root tissue was washed five times with sterile water. The treated rhizosphere soils and plant roots were stored in a refrigerator at −80°C for subsequent high-throughput sequencing.

### Soil Physical and Chemical Properties

The separated soils were placed in an oven and dried to a constant weight at 105°C. The water content of the soil (MC) was determined by the percentage of the weight of the soil lost water to the weight of the dried soil. The previously air-dried and sieved soil pH values were measured at (1:2.5 w/v) soil:water extraction ratio by the pH potentiometer. Soil electrical conductivity (EC) values were measured using an EC meter after shaking the soil:water (1:5 w/v) mixture. Total nitrogen (TN) and soil organic carbon (SOC) were analyzed using an elemental analyzer (Vario MACRO, CHNOS Elemental Analyzer, Elementar Co., Germany). Soils ranging in mass about 0.2–0.3 g were digested with nitric acid (HNO_3_), hydrofluoric acid (HF), hydrochloric acid (HCl), and perchloric acid (HClO_4_) (guaranteed reagent) (Sommers and Nelson, 1972). After the digestion, samples were centrifuged, decanted, diluted, and transferred quantitatively to centrifuge tubes, and total phosphorus (TP) was analyzed using an inductively coupled plasma optical emission spectroscopy (ICP-OES, Optima 7000 DV, PerkinElmer, United States). The total salt content of the soil was sent to an external laboratory (Inner Mongolia Hengtai Testing Technology Co., Ltd., China) to complete, including carbonate (CO_3_^2–^), bicarbonate (HCO_3_^–^), sulfate (SO_4_^2–^), chloride ion (Cl^–^), calcium ion (Ca^2+^), magnesium ion (Mg^2+^), and sodium ion and potassium ion (Na^+^ and K^+^). The total salt content was measured at least three times using the standard measurement method, and the measurement standard was referenced to the determination of the total water-soluble salt in the soil (NY/T 1121.16-2006). The measured values of physical and chemical factors are shown in Table 1. For more information on soil property, see Supplementary Table 1.

**TABLE 1:** The total salt and eight major ions in plant rhizosphere soil around the Ejinur Salt Lake.

**Sample point**	**TDS mg kg^–1^**	**CO_3_^2–^ mg kg^–1^**	**HCO_3_^–^ mg kg^–1^**	**Cl^–^ mg kg^–1^**	**SO_4_^2–^ mg kg^–1^**	**Ca^2+^ mg kg^–1^**	**Mg^2+^ mg kg^–1^**	**Na^+^ + K^+^ mg kg^–1^**
GA	40,190.4 ± 0.93b	0.0 ± 0.00b	427.5 ± 0.31b	18,168.2 ± 0.12b	7,781.2 ± 0.29a	260.6 ± 0.12a	2,041.2 ± 0.04a	11,511.7 ± 0.21b
GB	46,977.9 ± 1.20a	0.0 ± 0.00b	427.4 ± 0.28b	22,599.7 ± 0.38a	7,348.9 ± 0.35b	180.5 ± 0.13c	1,919.8 ± 0.08b	14,501.6 ± 0.30a
GC	18,189.0 ± 1.63c	0.0 ± 0.00b	275.2 ± 0.65c	8,331.1 ± 0.30c	3,314.4 ± 0.27c	240.6 ± 0.16b	887.1 ± 0.13c	5,140.7 ± 0.15c
GD	4,754.4 ± 1.89d	30.3 ± 0.26a	488.9 ± 0.67a	1,560.1 ± 0.30d	1,009.0 ± 0.14d	100.4 ± 0.13d	24.4 ± 0.11d	1,541.4 ± 0.43d

*Data are means ± standard deviation (*n* = 3). Values in the same column followed by the same letter(s) are not significantly different at *p* < 0.05. *Salicornia europaea* rhizosphere soils (GA), *Suaeda salsa* rhizosphere soils (GB), *Phragmites communis* rhizosphere soils (GC), and *Achnatherum splendens* rhizosphere soils (GD).*

### DNA Extraction and High-Throughput Sequencing

Microbial community genomic DNA was extracted from rhizosphere soils and root endophytic samples using the E.Z.N.A.^®^, soil DNA Kit (Omega Bio-tek, Norcross, GA, United States) according to the manufacturer’s instructions. The DNA extract was checked on 1% agarose gel, and DNA concentration and purity were determined with NanoDrop 2000 UV-vis spectrophotometer (Thermo Scientific, Wilmington, DE, United States). The hypervariable region V3–V4 of the bacterial 16S rRNA gene was amplified with primer pairs 338F (5′-ACT CCT ACG GGA GGC AGC AG-3′) and 806R (5′-ACT CCT ACG GGA GGC AGC AG-3′) (for rhizosphere soil bacterial community), and 799F (5′-AAC MGG ATT AGA TAC CCK G-3′) and 1193R (5′-ACG TCA TCC CCA CCT TCC-3′) (for endophytic bacterial community), respectively. ITS hypervariable regions of the fungal ITS rRNA gene were amplified with primer pairs ITS1F (5′-CTT GGT CAT TTA GAG GAA GTA A-3′) and ITS2R (5′-GCT GCG TTC TTC ATC GAT GC-3′) (for rhizospheric and endophytic fungal community). Briefly, PCR amplification had the following conditions: initial denaturation at 95°C for 3 min, denaturation at 95°C for 30 s, annealing at 55°C for 30 s, extension at 72°C for 45 s, a total of 40 cycles, and final extension at 72°C for 10 min; the experiment was repeated three times. The reaction product was detected by 2% agarose gel electrophoresis. The purified amplicons were paired-end sequenced (2 × 300 bp) on an Illumina MiSeq platform according to the standard protocols by Majorbio Bio-Pharm Technology Co., Ltd. (Shanghai, China). The raw sequencing data have been deposited into the National Center for Biotechnology Information (NCBI) Sequence Read Archive (SRA) database (Accession Number: PRJNA669550).

### Sequence Data Processing

The unique sequences among all reads were used to define operational taxonomic units (OTUs) using UPARSE version 7.1 with 97% sequence similarity (Edgar, 2013). In order to compare different samples at the same sequencing level, the samples were flattened according to the minimum number of sample sequences (Fang et al., 2018), and the representative sequences in each OTU of bacteria and fungi were compared with the Silva132 and 16S rRNA bacteria and Unite7.2 and ITS rRNA fungi database (Wang et al., 2007). The sample rarefaction curve was used to evaluate the sequencing depth of the high-throughput sequencing results of bacteria and fungi in eight groups of rhizosphere soil samples and eight groups of plant root samples. The evaluation results are shown in Supplementary Files and Supplementary Figure 1. At the same time, the alpha diversity (Shannon index and Sobs index) of each group of samples was analyzed, and species richness analysis in 16 groups of samples was performed.

### Statistical Analyses

All data were analyzed by SPSS 17.0 software, and the data were reflected as the mean ± standard deviation (SD) based on three replicates. The statistical significance of the difference between the means was performed by one-way analysis of variance (ANOVA). Significance was calculated by Duncan’s multiple range test at the *p* < 0.05 level.

The results of microbial *α*-diversity were drawn by using Origin 2017 software. Regression analysis was used to show linear correlation between plant species (PBI) and soil environmental factors and β-diversity (PC1 axis value in PCoA results based on Bray–Curtis) of different rhizocompartments of microorganisms (details are in the Supplementary Materials). Distance-based redundancy analysis (db-RDA) was used to analyze the microbial community structure and the relationship between the microbial communities and environmental factors based on Bray–Curtis (McArdle and Anderson, 2001). The environmental factors included SOC, TP, TN, TDS, CO_3_^2––^, HCO_3_^––^, SO_4_^2––^, Cl^––^, and Ca^2+^. Linear discriminant analysis effect size (Lefse) was performed online^1^. The logarithmic LDA score was set to 4.0 with statistical significance (*p* < 0.05). LEfSe was conducted to identify taxa that display significant differences from phylum to genus level in the rhizosphere soil and root endophytic samples. Microbial correlation networks were constructed to explore separately the interactions of bacteria and fungi from rhizospheric and root endophytic samples. The bacterial and fungal networks showed different patterns of correlation based on significant correlations (Spearman’s correlation coefficients, *| r|* > 0.6, *p* < 0.01). Networks were visualized in Cytoscape 3.4.0 (Shannon et al., 2003). PICRUSt2 was used to predict the bacterial and fungal metabolic pathways based on MetaCyc database (Caspi et al., 2020).

## Results

### Analysis of Soil Physicochemical Parameters

From the edge of the Ejinur Salt Lake to its periphery, the degree of soil salinization decreased gradually and plants showed obvious zonal distribution characteristics [the dominant plants were *S. europaea* (GA), *S. salsa* (GB), *P. communis* (GC), and *A. splendens* (GD)]. Rhizosphere soil samples in this study were all alkaline soil (pH was from 7.64 to 8.47) (Supplementary Table 1). Table 1 shows the total salt content and eight major ion contents of rhizosphere soil samples. One-way ANOVA analysis shows that the total salt content and eight major ion contents of rhizosphere soil changed significantly. Soil vegetation covers were closely related to the soil parameters. For instance, *S. europaea* rhizosphere soils were characterized with relative high concentrations of SO_4_^2–^, Ca^2+^, Mg^2+^ (Table 1), TN, and MC (Supplementary Table 1). *S. salsa* rhizosphere soils featured relatively high TDS, Cl^–^, Na^+^, and K^+^ (Table 1), SOC, TP, and EC levels (Supplementary Table 1), while *A. splendens* rhizosphere soils had high levels of CO_3_^2–^, HCO_3_^–^, and pH (Table 1 and Supplementary Table 1).

### Microbial Community Composition and Their α-Diversity

**FIGURE 1:**
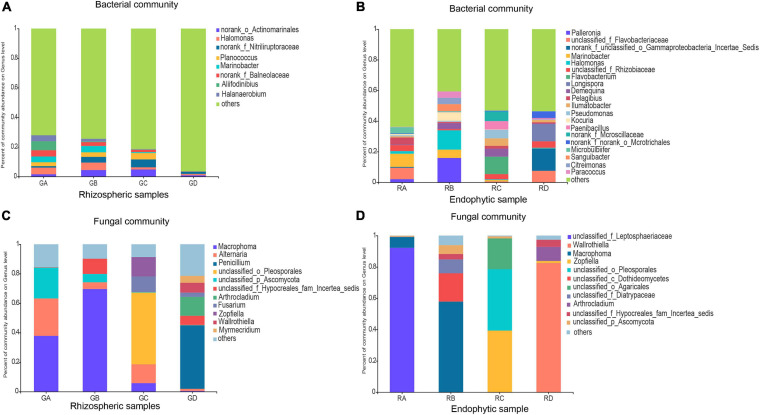
Effects of rhizosphere soil and root endophytic samples on the community composition at the genus level of Bacteria and Fungi in the Ejinur Salt Lake. Genus with relative abundance less than 4% were defined as others. **(A)** Bacterial rhizospheric samples, **(B)** bacterial endophytic samples, **(C)** fungal rhizospheric samples, and **(D)** fungal endophytic samples. *Salicornia europaea* **(A)**, *Suaeda salsa* **(B)**, *Phragmites communis* **(C)**, and *Achnatherum splendens* **(D)**.

Based on 97% sequence similarity, the bacterial and fungal sequences of rhizosphere soil samples were aggregated into 6,497 and 996 OTUs, and the bacterial and fungal sequences of root endophytic samples were aggregated into 1,153 and 279 OTUs. The rarefaction curve for observing the rhizosphere soil and the endophytic bacteria and fungi OTUs reached a plateau, indicating that sequencing effort was sufficient to represent the entire bacterial and fungal diversity (Supplementary Figure 1). In filtered high-throughput sequences, the results demonstrated that the composition of major bacterial phylum or sub-phylum in rhizosphere soil and root endophytic samples was not the same, of which 12 bacterial and 3 fungal major phyla in rhizosphere soil samples accounted for more than 95%. For root endophytic samples, seven bacterial and two fungal major phyla accounted for >95%. Proteobacteria (31.55–40.20%) and Actinobacteria (7.99–35.35%) were the major bacterial groups in rhizosphere soil, representing more than 40.00% of the bacterial sequences. The bacterial groups in the rhizosphere soil were Bacteroidetes, Firmicutes, Chloroflexi, Gemmatimonadetes, Patescibacteria, Acidobacteria, Deinococcus-Thermus, Halanaerobiaeota, Planctomycetes, and Cyanobacteria (Supplementary Figure 2). For the root endophytic samples, Proteobacteria (49.63–64.56%) and Actinobacteria (5.78–32.01%) were mainly the predominant bacterial groups, representing more than 60.00% of the bacterial sequences. Bacteroidetes, Firmicutes, Planctomycetes, Chloroflexi, and Fibrobacteres were bacterial groups in root endophytic samples (Supplementary Figure 2). Ascomycota (94.27–96.77%) was the most abundant fungal phylum in rhizosphere soil followed by Basidiomycota (2.10–5.51%) (Supplementary Figure 2). In contrast, the most abundant fungal phylum of the endosphere was Ascomycota, which accounted for 72.65–99.98%, followed by Basidiomycota, which accounted for 0.00–27.32% (Supplementary Figure 2). The main microbial communities of rhizosphere soil and root endophytic samples are shown in Figure 1, and their composition at the genus level was also not the same. The relative abundances of the main genera are shown in Supplementary Table 2.

**FIGURE 2:**
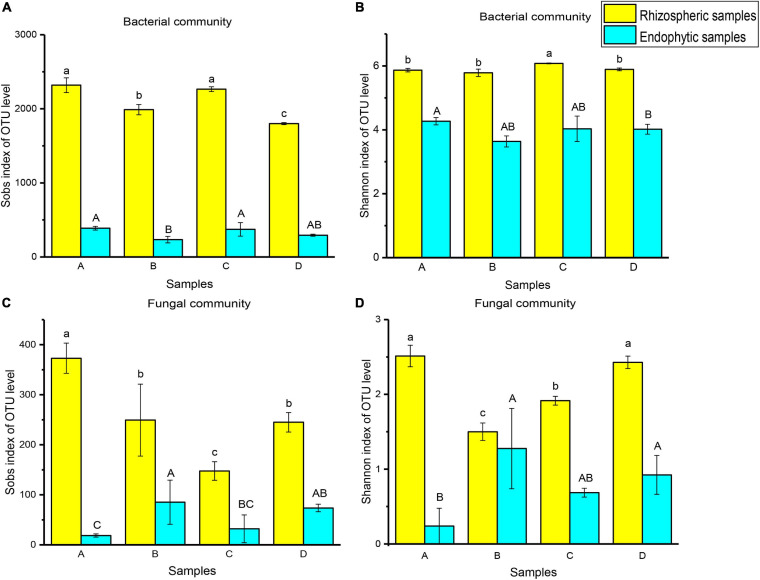
Ejinur Salt Lake different rhizosphere soil and root endophytic samples of microbial α-diversity. Different uppercase (Endophytic samples) and lowercase (Rhizospheric samples) letters indicate significant differences among plant species (*p* < 0.05). The abscissa letters indicate the plant species [*S. europaea* **(A)**, *S. salsa* **(B)**, *P. communis* **(C)**, and *A. splendens* **(D)**].

To compare the significant differences in microbial diversity between samples, α-diversity was represented by statistical methods (Figure 2). The microbial communities belonged to rhizosphere soil and root endophytic samples. Among them, the observed value of bacteria is the highest, followed by fungi. In addition, it was demonstrated that the abundance and diversity of microorganisms in rhizosphere soil were higher than that of root endophytic samples. For bacterial community, the microbial richness of rhizospheric samples A (2319 ± 99.35) and endophytic samples A (389.33 ± 25.42) were the higher. The rhizospheric sample C (6.08 ± 0.01) and endophytic sample A (4.27 ± 0.11) had the higher microbial diversity. For fungal community, the microbial richness (373 ± 30.05) and diversity (2.51 ± 0.14) of rhizospheric sample A were higher. Endophytic sample B had higher microbial richness (85.33 ± 40.02) and diversity (1.28 ± 0.54) (Figure 2 and Supplementary Table 3).

### Distance-Based Redundancy Analysis and Variance Distribution Analysis of Microbial Communities and Soil Characteristics

In both rhizospheric samples and endophytic samples, the plant species were positively correlated with the *β*-diversity of the microbial communities (Supplementary Figures 3–6). Regression analysis showed that the PC1 axis value in the PCoA results increased with increasing CO_3_^2–^ (*r* = 0.957, *p* < 0.0001) in bacterial rhizospheric samples. The PC1 axis value decreased with increasing TP (*r* = 0.895, *p* < 0.001), SOC (*r* = 0.815, *p* < 0.01), TDS (*r* = 0.916, *p* < 0.0001), SO_4_^2–^ (*r* = 0.933, *p* < 0.0001), Cl^–^ (*r* = 0.912, *p* < 0.0001), and Ca^2+^ (*r* = 0.820, *p* < 0.01) in bacterial rhizospheric samples. For bacterial endophytic samples, TP (*r* = 0.952, *p* < 0.0001), SOC (*r* = 0.947, *p* < 0.0001), TDS (*r* = 0.974, *p* < 0.0001), SO_4_^2–^ (*r* = 0.942, *p* < 0.0001), Cl^–^ (*r* = 0.979, *p* < 0.0001), and Ca^2+^ (*r* = 0.592, *p* < 0.05) were negatively correlated with microbial β-diversity, while CO_3_^2–^ (*r* = 0.851, *p* < 0.001) was positively correlated with microbial β-diversity. For fungal community, the PC1 axis value decreased with increasing TP (*r* = 0.860, *p* < 0.001), SOC (*r* = 0.777, *p* < 0.01), TDS (*r* = 0.884, *p* < 0.001), SO_4_^2–^ (*r* = 0.906, *p* < 0.0001), Cl^–^ (*r* = 0.879, *p* < 0.001), and Ca^2+^ (*r* = 0.856, *p* < 0.001) in fungal rhizospheric samples. Similarly, the PC1 axis value decreased with increasing TP (*r* = 0.940, *p* < 0.0001), SOC (*r* = 0.789, *p* < 0.01), TDS (*r* = 0.938, *p* < 0.0001), SO_4_^2–^ (*r* = 0.979, *p* < 0.0001), Cl^–^ (*r* = 0.924, *p* < 0.0001), and Ca^2+^ (*r* = 0.736, *p* < 0.01) in fungal endophytic samples. Moreover, the PC1 axis value increased with increasing CO_3_^2–^ (*r* = 0.973, *p* < 0.0001) in fungal rhizospheric samples, while the PC1 axis value was not significantly correlated with CO_3_^2–^ in the fungal endophytic samples.

**FIGURE 3:**
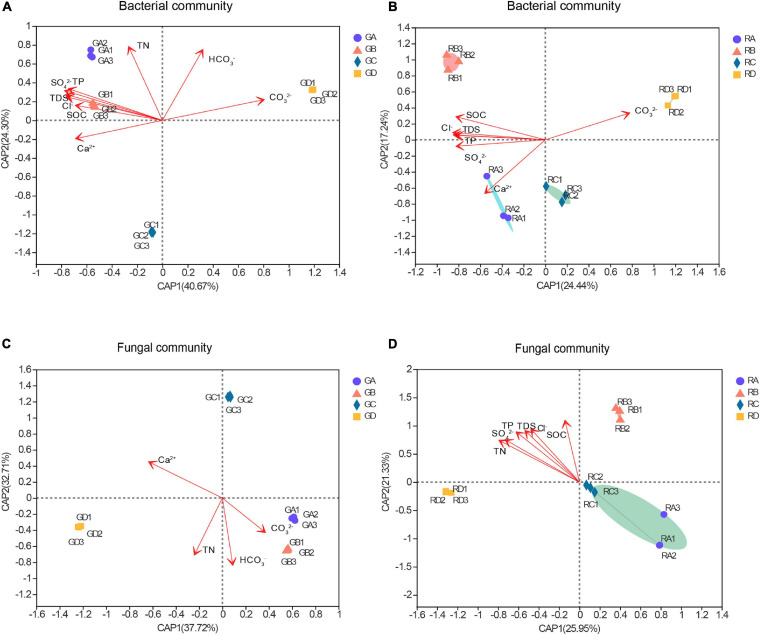
The distance-based redundancy analysis (db-RDA) of these microbial community structure and environmental parameters. Numbers in parentheses in the axis labels was a percentage explanatory variable. **(A)** Bacterial rhizospheric samples, **(B)** bacterial endophytic samples, **(C)** fungal rhizospheric samples, and **(D)** fungal endophytic samples. *S. europaea* **(A)**, *S. salsa* **(B)**, *P. communis* **(C)**, and *A. splendens* **(D)**.

The distance-based redundancy analysis (db-RDA) was used to reflect the relationship between microbial structure and soil characteristics (Figure 3). The results showed that the significant differences in microbial community structure at the OTU level and the salinity gradient could influence the rhizospheric and endophytic microbial structure (Figures 3A–D). The microbial structure could be determined according to the size of the angle between bacterial or fungal and environmental factors and the length of the connection in distance-based redundancy analysis. Among all the environmental parameters, the SO_4_^2–^ (*R*^2^ = 0.9963, *p* = 0.001) had a significant influence on the rhizosphere bacterial communities (Figure 3A), while the SOC (*R*^2^ = 0.9848, *p* = 0.001) had the greatest effect on endophytic bacterial communities (Figure 3B). For the fungal communities, the HCO_3_^–^ (*R*^2^ = 0.9906, *p* = 0.001) and SOC (*R*^2^ = 0.6906, *p* = 0.007) were significantly related to the rhizospheric fungal communities and the endophytic fungal communities, respectively (Figures 3C,D). Overall, the influence of environmental factors on bacterial communities were significantly greater than that of fungal communities, and the results showed that bacteria are more widely affected by soil characteristics.

### LEfSe Analysis to Reveal Dominant Microbial Taxa in Different Rhizocompartments

**FIGURE 4:**
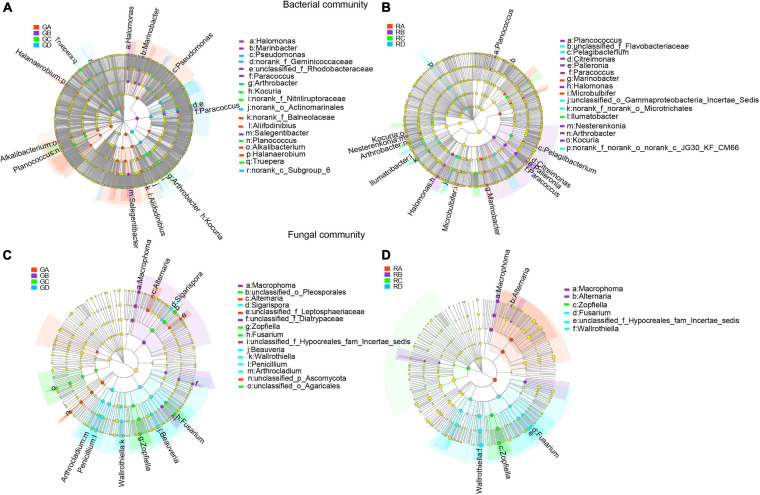
The differential phylogenetic distribution of bacteria **(A,B)** and fungi **(C,D)** in Ejinur Salt Lake different rhizosphere soil and root endophytic samples. The linear discriminant analysis scores of >4. The five rings of the cladogram stand for phylum (innermost), class, order, family, and genus. Different colored nodes represent microorganisms that play an important role in the grouping represented by color; yellow represents non-significant. Uppercase “G” represents the rhizospheric sample, and uppercase “R” represents the endophytic samples. *S. europaea* **(A)**, *S. salsa* **(B)**, *P. communis* **(C)**, and *A. splendens* **(D)**.

LEfSe was used to evaluate the different taxa from rhizosphere soil and root endophytic samples. The results showed that the number of dominant bacteria and fungi were 18 and 15 in rhizospheric samples (Figures 4A,C), respectively, while the number of dominant endophytic bacteria and fungi were 16 and 6 in endophytic samples (Figures 4B,D), respectively. This study indicated that whether it was bacterial communities or fungal communities, the rhizosphere soil exhibited a greater number of dominant genera than that in the endophytic environments. Along the salinity gradient decreasing, *Aliifodinibius*, *Alkalibacterium*, *Halanaerobium* (GA), *Halomonas*, *Marinobacter, Paracoccus*, *Salegentibacter* (GB), *Kocuria*, *Planococcus*, *Truepera* (GC), and *Pseudomonas* and *Arthrobacter* (GD) were the dominant taxa at the genus level of rhizosphere bacterial communities (Figure 4A). The dominant taxa in the endophytic bacterial communities were *Marinobacter*, *Microbulbifer* (RA), *Planococcus*, *Citreimonas*, *Palleronia*, *Paracoccus*, *Halomonas*, *Nesterenkonia*, *Arthrobacter*, *Kocuria* (RB), *Ilumatobacter* (RC), and *Pelagibacterium* (RD) (Figure 4B). For the fungal communities, *Alternaria* (GA), *Macrophoma* (GB), *Zopfiella*, *Fusarium* (GC), and *Sigarispora*, *Beauveria*, *Wallrothiella*, *Penicillium*, and *Arthrocladium* (GD) were the dominant genus in the rhizosphere fungal communities (Figure 4C), and *Macrophoma*, *Alternaria* (RB), *Zopfiella* (RC), and *Fusarium* and *Wallrothiella* (RD) were the dominant taxa in the endophytic fungal communities (Figure 4D). Interestingly, the dominant species in the root were the same as those in rhizosphere soil for the fungal communities.

### Correlation Molecular Network Analysis of Microbial Communities

**FIGURE 5:**
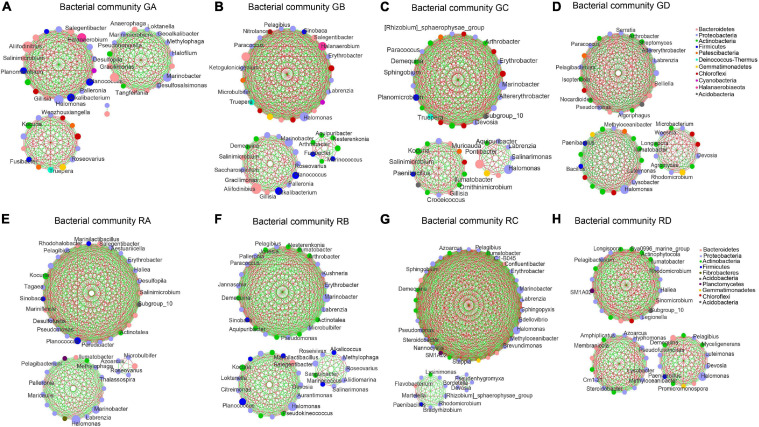
An overview of the bacterial network interactions in Ejinur Salt Lake different rhizosphere soil and root endophytic samples. The connections in the network represent a strong (| *r*| > 0.6) and significant (*p* < 0.01) correlation. Colors of nodes represent different major phyla; the node representing genus is written inside. A green link indicates a negative interaction, whereas a red link implies a positive interaction between two individual nodes. Uppercase “G” represents the rhizospheric sample, and lowercase “R” represents the endophytic samples. **(A–D)** *S. europaea* rhizosphere soil (GA), *S. salsa* rhizosphere soil (GB), *P. communis* rhizosphere soil (GC) and *A. splendens* rhizosphere soil (GD). **(E–H)** Salicornia europaea endophytic samples (RA), Suaeda salsa endophytic samples (RB), Phragmites communis endophytic samples (RC) and Achnatherum splendens endophytic samples (RD).

The correlation microbial networks were explored microbial interactions in rhizosphere soil and root endophytic samples (Figure 5 and Supplementary Figure 7). The results showed that there were differences in the interaction between bacterial and fungal taxa under a salinity gradient (Supplementary Tables 4, 5). For endophytic samples of bacteria, the number of negative links from RA to RC was lower than RD (Supplementary Table 4). Except for the *A. splendens* sample (RD), whether bacteria or fungi, the positive links between endophytes were greater than those between rhizosphere soil microorganisms (Supplementary Tables 4, 5). The number of positive links in fungal taxa was far greater than the number of negative links (Supplementary Table 5). Interestingly, the same genus (e.g., *Kocuria* in the *A. splendens* sample) was observed in rhizosphere soil and root endophytic samples. It implied that fungal taxa might cooperate more to adapt to high salinity niches. In addition, Proteobacteria were defined as the core microbial taxa of GA–GC samples based on its abundance and close connections with other taxa. It accounted for 40.20, 36.03, and 31.55% of GB, GA, and GC samples, respectively. The core bacterial taxa of GD were Actinobacteria (35.55%) (Figures 5A–D). The core bacterial taxa of RA–RD were primarily Proteobacteria. It accounted for 64.56, 58.50, 52.10, and 49.63% of RA, RB, RD, and RC, respectively (Figures 5E–H). Whether in the rhizosphere fungal communities or the endophytic fungal communities (Supplementary Figure 7), Ascomycota were core fungal taxa (>72%). Moreover, the bacterial and fungal 10 metabolic pathways in rhizospheric and endophytic samples were predicted by PICRUSt2. The differences in the average relative abundance of different metabolic pathways are shown in Supplementary Tables 8, 9.

## Discussion

### Drivers of Microbiome Niche Differentiation

Soil salinity can affect not only the distribution, composition, and diversity of plant communities, but also the diversity and community structure of microbial in soil (Xi et al., 2016; Zhang et al., 2019). In the study, the high-throughput sequencing technique was used to reveal the diversity and abundance of bacterial and fungal communities in rhizosphere soil samples and root endophytic samples under different salinity. The study showed that the bacterial and fungal communities in rhizosphere soil were more abundant than root endophytic microbial communities, which is consistent with previous studies (Lu et al., 2016; Li et al., 2018). Based on the significant difference of microbial composition at the phylum level (Supplementary Tables S6 and S7), microbial α-diversity (Figure 2) and other results (Figures 3–5), it indicates that a strong habitat filtering mechanism that may affect the microbial composition from different rhizocompartments of plants (Qin et al., 2016). These filtering effects have previously been reported in the different rhizocompartments of soya and alfalfa (Xiao et al., 2017) and the rhizosphere and endosphere of *Arabidopsis thaliana* (Lundberg et al., 2012). This filtration was related to the physical and chemical characteristics of the rhizosphere soil and the change from the soil to the endosphere environment (Gottel et al., 2011). Moreover, previous works have found that the richness and evenness of microbial communities decreased with the increase of salt-rich soil and sediment (Wang et al., 2010; Zhao et al., 2018). However, some studies have shown that bacterial and fungal communities are positively correlated with salinity in a saline–alkali environment, and the existence of plants will increase the diversity of microorganisms independently of salinity (Foti et al., 2008; Vanegas et al., 2019). In this study, our investigation found that the degree of soil salinization decreased gradually and dominant plants showed obvious zonal distribution characteristics from the edge of the lake to the periphery. Moreover, there were also significant differences in soluble salt ion concentrations and soil nutrients under different salinity in the littoral zones of Ejinur Salt Lake (Table 1). The difference in soil properties and plant species may result in the difference of microbial community composition and structure. Although salinity plays an important role in the construction of bacterial and fungal communities, plant species and other soil parameters (e.g., soil pH value or soil nutrient content) may also affect the bacterial and fungal diversity and community composition from different rhizocompartments in soil environments of salt lakes littoral zones.

An additional analysis of significant difference in community structure were identified among different rhizocompartments (Figure 3). This finding was consistent with previous studies on the microbial communities in different root chambers of tomato in different geographical locations. For endophytic microbial communities, the high difference of community structure may depend on the inherent immune system of plants, while rhizosphere soil microbes are less affected by host-dependent selection (Lee et al., 2019). The growth of host plants may be affected by the soil environments. Therefore, the high difference of endophytic microorganisms depends on the plant and other soil physicochemical properties. In addition, the microbial composition related to salinity, soluble salt ion concentration, and soil nutrients (Lozupone and Knight, 2007; Barin et al., 2015). Our regression analysis results confirmed the inference that rhizosphere soil microorganisms and root endophytic microorganisms are affected by plants, soil nutrients, and different soluble salt ions (Supplementary Figures 3–6). This was also consistent with our db-RDA results that show that rhizosphere soil and root endophytic microbial communities have significant relationships with soil salinity, different soluble salt ions, and soil nutrients (Figure 3). The SOC in soil nutrients was overall an important driving factor for the microbial community structure in the whole range of the salinity gradient, because SOC can mitigate the salinity pressure in microorganisms. Microorganisms may produce some compounds such as osmotic regulators (e.g., glutamine and proline) to cope with salt stress (Barin et al., 2015). The synthesis of these compounds depletes a lot of energy, which is obtained by the decomposition of organic compounds (Oren, 1999).

The microbial interaction is also very important for exploring the structure and dynamics of soil bacterial and fungal communities (Faust and Raes, 2012; Xue et al., 2017). It is possible to determine the positive and negative co-occurrence correlation between bacterial and fungal members and further reflect the functional relationship between synergy and antagonism (Benidire et al., 2020). Our results showed that bacterial hubs were mainly members of Proteobacteria and Actinomycota, such as *Labrenzia* and *Kocuria*. PGPR strains belonging to genera *Kocuria* have been proven to indirectly improve the salt tolerance of maize (Li et al., 2020). A strain isolated from the genus *Labrenzia* in the rhizosphere of the halophyte *Arthrocnemum macrostachyum* has been verified to have halophilic properties (Camacho et al., 2016). Moreover, the root endophytic fungi played a certain role in assisting plants to adapt to the salt environments (Chadha et al., 2015). The composition of the genera among different modules were not the same, which further reflects the unique ecological characteristics of these combinations. The aggregation patterns of these microorganisms showed that the interaction between species and environmental filtration plays a core role in the formation of community structure. In the results, except for the *A. splendens* sample, whether bacteria or fungi, the positive links between endophytes were greater than those between rhizosphere soil microorganisms (Supplementary Tables 4, 5). Generally speaking, positive connections mean cross-feeding, while negative relationships represent competition in the network (Zheng et al., 2017). The pMENs analysis of Wang et al. (2019) found that salinity and alkalinity significantly increased microbial interactions from different cadmium-contaminated soils. Therefore, it can be inferred that the regulation of microbial interaction may be a strategy for microorganisms to deal with strong salt stress. Previous study showed that the hubs involved in positive interactions under certain conditions may behave differently under stress conditions and mainly establish antagonistic interactions. The results may be related to the expression of genes involved in stress tolerance (Benidire et al., 2020). The study found that in the network structure, some members of the hubs belonged to *Neocamarosporium* and *Aspergillus* (Supplementary Figures 5C,D). They mainly participated in the collaborative interaction only under salt stress, while they showed antagonistic interaction under non-salt stress. Some genera in the endophyte network structure were found to occur in the network structure of rhizosphere soil. The results also found that the number of OTUs and microbial diversity of rhizosphere soil was much larger than that of root endophytic samples. These results suggested that endophytes may be a part of rhizosphere microbes. Tian et al. (2015) also indicated that tomato endophytic microbiota was obtained from rhizosphere microbes.

### Dominant Microbiome Within Different Rhizocompartments

According to the LEfSe analysis (LDA > 4.0), the microbial taxa shown in the results (Figure 4) were defined as the dominant taxa (Richa et al., 2017; Zhao et al., 2020; Zhang et al., 2021). These dominant taxa can be used as indicator species for different plants around the salt lake and may assist plants to adapt to unfavorable environments. For example, *Marinobacter*, *Palleronia*, *Ilumatobacter*, and *Arthrobacter* were the dominant genera in the samples of *S. europaea*, *S. salsa*, *P. communis*, and *A. splendens*, respectively. Previous works have shown that *Marinobacter* and *Palleronia* have good endurance in a broad range of salinity with exceptional adaptation capacities (Martinez-Checa et al., 2005; Li et al., 2019). *Ilumatobacter* and *Arthrobacter* are beneficial taxa, and PGPR strains belonging to genera *Arthrobacter* can promote plant growth, increase plant salt tolerance, and effectively improve agricultural productivity (Kumar et al., 2019; Zhao et al., 2019; Stassinos et al., 2021). *Alternaria*, *Macrophoma*, *Zopfiella*, and *Penicillium* were the dominant fungal taxa with high abundance in plant samples of *S. europaea*, *S. salsa*, *P. communis*, and *A. splendens*, respectively. In previous studies, *Alternaria* and *Macrophoma* have been found in the salt crusts and roots of halophytes (You et al., 2012; Liu et al., 2014). These genera are known plant pathogens that can cause leaf spots and fusarium wilt (Chaerani and Voorrips, 2006; Jeyaraman and Robert, 2017). *Penicillium* has been shown to confer soybean resistance to salt stress (Khan et al., 2011). Moreover, microbial metabolic functions also had a certain positive effect on salt stress. Yu et al. (2021) found that Maize bHLH55 functions regulated the biosynthesis of ascorbic acid by directly regulating GDP-mannose pathway genes and thus play a positive role in salt tolerance. Therefore, these dominant microbial taxa had been proven to improve abiotic stress, promote plant growth, and confer plants the ability to resist salt stress.

Previous studies also found that the bacterial communities were more sensitive to the soil environments. The research results confirmed that dominant taxa of bacterial communities in rhizosphere soil or the root endosphere are significantly more than those of fungal communities. Bacteria generally is smaller and has a shorter life cycle compared with fungi (Chen et al., 2020), making them more susceptible to external interference and causing them to be more sensitive. The rhizosphere soil microbial communities showed higher sensitivity than the root endophytic microbial communities, containing more biomarkers. The rhizosphere has the most direct resource exchange interface between the soil environments and the root systems (Yu and Hochholdinger, 2018) and is more easily affected by the soil environments than the root endosphere. These results indicated that improving plant tolerance to salt stress depends on not only the interaction of the entire microbial communities but also some specific microbial consortia (de Zelicourt et al., 2013). Exploring the interaction between microorganisms and the dominant taxa may be beneficial to understand the salt tolerance of plants and the importance of related microorganisms. In short, exploring the interaction between microorganisms and excavating the potential functional roles in dominant taxa are beneficial for understanding the interaction mechanism between plants and related microorganisms in the littoral zones of salt lakes. Although the findings of this study expand our understanding of the difference in bacterial and fungal community and their interactions from rhizosphere and endosphere of four dominant plants in the littoral zones along the salinity gradient, there is still much to be learned about the special roles of functional microorganisms and their relationships. Therefore, it should be further explored by using advanced sequencing technology (e.g., metagenomics and multiomics) in the future.

## Conclusion

This study demonstrated the microbial community composition in different rhizocompartments of four dominant plants around the Ejinur Salt Lake and revealed the main driving factors affecting the microbial community composition. The bacterial and fungal richness and phylogenetic diversity did not increase with the decrease of salinity, indicating that salinity may not be the main driving factor for microorganisms from different rhizocompartments of plants. Regression analysis indicated that rhizospheric and endophytic microorganisms were affected by soil nutrients and different soil soluble salt ions and plant species. The db-RDA future revealed that bacteria from rhizospheric samples were the most significantly affected by SO_4_^2–^, while fungi were the most significantly affected by HCO_3_^–^. For endophytic samples, both bacteria and fungi were the most significantly affected by SOC. In addition, the correlation network analysis indicated that the interaction mode between microorganisms has potential beneficial effects in regulating salt stress. LEfSe analysis further revealed that there were dominant microbial taxa under soil environments of salinity gradient, and those microbial taxa had a positive response to plant species. This study provided new insights for in-depth understanding of the roles of microorganisms from different rhizocompartments (rhizosphere and endosphere), and it was of great significance for excavating the potential microbial resources resistant to saline–alkali stress in the littoral zones of salt lakes.

## Data Availability Statement

The datasets generated for this study can be found in online repositories. The names of the repository/repositories and accession number(s) can be found below: https://www.ncbi.nlm.nih.gov/, PRJNA669550.

## Author Contributions

JL: writing—original draft, investigation, methodology, validation, visualization, data analysis, and software. ZZ: writing—review and editing, data curation, visualization, formal analysis, and software. YH: investigation, resources, and methodology. FD: software and data curation. BH and LW: methodology. ZB: conceptualization. WG: writing—review and editing, supervision, funding acquisition, and project. All authors contributed to the article and approved the submitted version.

## Conflict of Interest

The authors declare that the research was conducted in the absence of any commercial or financial relationships that could be construed as a potential conflict of interest.

## Footnotes

1 https://cloud.majorbio.com
