# Supplementary material for: Exploring Microbial Resource of Different Rhizocompartments of Dominant Plants Along the Salinity Gradient Around the Hypersaline Lake Ejinur
Source: Front Microbiol. 2021 Jul 12;12:698479. doi: 10.3389/fmicb.2021.698479 (PMC8312270; doi:10.3389/fmicb.2021.698479)
Supplement: Supplementary file 1 [file Data_Sheet_1.docx]

**Supplemental Materials**

**Supplementary Figures and Tables**:

1 Supplementary Text

2 Supplementary Tables

3 Supplementary Figures

**Supplementary Text:**

**Statistical analyses:** Regression analysis is a statistical analysis method to determine the relationship between one or more independent variables and dependent variables. Regression analysis of environmental factors and *β*-diversity, according to the PCoA analysis results, the score of each sample on the PC1 axis was used as the y-axis, and the environmental factors corresponding to the sample was used as the x-axis to make a scatter plot and perform regression analysis in this study. The environmental factors included plant species (PBI) and soil physicochemical factors (TP, TN, SOC, TDS, SO_4_^2-^, HCO_3_^-^, CO_3_^2-^, Cl^-^, Ca^2+^).

**Table S1.** The residual physicochemical parameters in plant rhizosphere soil around Ejinur Salt Lake.

| Sample  Point | SOC  mg kg^-1^ | TN  mg kg^-1^ | TP  mg kg^-1^ | pH | EC  ds m^-1^ | MC  % |
| --- | --- | --- | --- | --- | --- | --- |
| GA | 860.00±5.42b | 811.71±12.55a | 833.98±16.10b | 7.90±0.01b | 11.50±0.02b | 20.21±2.79a |
| GB | 1254.05±5.45a | 768.69±9.33b | 867.29±14.22a | 7.73±0.01c | 14.34±0.03a | 17.17±0.85ab |
| GC | 659.82±5.07c | 246.77±8.83d | 606.67±15.62c | 7.64±0.02d | 6.21±0.01c | 15.89±0.68b |
| GD | 433.48±6.10d | 583.47±8.87c | 513.72±22.84d | 8.47±0.02a | 1.84±0.00d | 9.55±1.36c |

Data are means ± standard deviation (n=3). Values in the same column followed by the same letter(s) are not significantly different at *P* < 0.05. *Salicornia europaea* rhizosphere soils (GA), *Suaeda salsa* rhizosphere soils (GB), *Phragmites communis* rhizosphere soils (GC) and *Achnatherum splendens* rhizosphere soils (GD).

**Table S2.** The main genera of bacteria and fungi from rhizospheric samples and endophytic samples and their relative abundance.

|  | Genus | A | B | C | D |
| --- | --- | --- | --- | --- | --- |
| Bacterial rhizosphere | Halomonas | 4.55% | 5.04% | 1.49% | 0.92% |
|  | Planococcus | 2.66% | 3.08% | 4.33% | 0.05% |
|  | Marinobacter | 3.90% | 4.29% | 0.65% | 0.06% |
|  | Aliifodinibius | 6.06% | 0.50% | 0.29% | 0.00% |
|  | Halanaerobium | 4.01% | 1.75% | 0.25% | 0.01% |
| Bacterial endosphere | Palleronia | 2.07% | 15.88% | 0.00% | 0.01% |
|  | Marinobacter | 8.66% | 5.48% | 0.89% | 0.07% |
|  | Halomonas | 1.66% | 12.60% | 0.43% | 0.37% |
|  | Flavobacterium | 0.00% | 0.00% | 11.33% | 0.00% |
|  | Longispora | 0.00% | 0.00% | 0.00% | 11.15% |
|  | Demequina | 0.00% | 3.84% | 5.17% | 0.00% |
|  | Pelagibius | 4.56% | 0.00% | 0.00% | 0.00% |
|  | Ilumatobacter | 0.00% | 0.00% | 4.95% | 1.60% |
|  | Pseudomonas | 0.00% | 0.00% | 5.59% | 0.00% |
|  | Kocuria | 0.00% | 5.66% | 0.00% | 0.00% |
|  | Paenibbacillus | 0.00% | 0.00% | 5.52% | 0.00% |
|  | Microbulbifer | 4.11% | 0.00% | 0.00% | 0.00% |
|  | Sanguibacter | 0.00% | 4.60% | 0.00% | 0.00% |
|  | Citreimonas | 0.00% | 4.14% | 0.00% | 0.00% |
|  | Paracoccus | 0.00% | 4.11% | 0.00% | 0.00% |
| Fungal rhizosphere | Macrophoma | 37.81% | 69.61% | 5.74% | 0.47% |
|  | Alternaria | 25.32% | 4.56% | 12.77% | 1.44% |
|  | Penicillium | 0.02% | 0.01% | 0.00% | 43.09% |
|  | Arthrocladium | 0.00% | 0.00% | 0.00% | 12.79% |
|  | Fusarium | 0.06% | 0.01% | 10.61% | 2.86% |
|  | Zopfiella | 0.00% | 0.00% | 13.16 | 0.10% |
|  | Wallrothiella | 0.00% | 0.00% | 0.00% | 6.65% |
|  | Myrmecridium | 0.00% | 0.00% | 0.00% | 4.68% |
| Fungal endosphere | Wallrothiella | 0.00% | 0.00% | 0.00% | 82.44% |
|  | Macrophoma | 6.87% | 57.86% | 0.01% | 0.04% |
|  | Zopfiella | 0.00% | 0.00% | 39.35% | 1.32% |
|  | Arthrocladium | 0.00% | 0.00% | 0.00% | 9.00% |

The letters indicate the plant species (*Salicornia europaea* (A), *Suaeda salsa* (B), *Phragmites communis* (C) and *Achnatherum splendens* (D)).

**Table S3.** Alpha diversity indexes of bacterial and fungal community based on 97% OTU in the rhizospheric samples and endophytic samples.

|  | **Samples** | **Bacterial community** | | **Fungal community** | |
| --- | --- | --- | --- | --- | --- |
|  |  | **Sobs** | **Shannon** | **Sobs** | **Shannon** |
| Rhizospheric samples | A | 2319.00±99.35 a | 5.87±0.06 b | 373.00±30.05 a | 2.51±0.14 a |
|  | B | 1988.30±69.58 b | 5.78±0.11 b | 249.33±71.91 b | 1.50±0.11 c |
|  | C | 2265.30±32.39 a | 6.08±0.01 a | 147.67±18.50 c | 1.92±0.06 b |
|  | D | 1800.30±14.36 c | 5.89±0.04 b | 245.00±19.52 b | 2.43±0.08 a |
| Endophytic samples | A | 389.33±25.42 A | 4.27±0.11 A | 18.67±3.21 C | 0.24±0.29 B |
|  | B | 234.33±43.47 B | 3.64±0.17 A | 85.33±44.02 A | 1.28±0.54 A |
|  | C | 372.67±91.35 A | 4.03±0.40 B | 32.00±27.73 BC | 0.69±0.06 AB |
|  | D | 293.33±13.32 AB | 4.02±0.15 A | 73.67±7.57 AB | 0.92±0.26 A |

Different letter indicates significances according to Duncan's multiple range test at the *P*<0.05 level using a one-way ANOVA. Different uppercase (Endophytic samples) and lowercase (Rhizospheric samples) letters indicate significant differences among plant species (*p* < 0.05). The letters indicate the plant species (*Salicornia europaea* (A), *Suaeda salsa* (B), *Phragmites communis* (C) and *Achnatherum splendens* (D)).

Sobs indicate richness; Shannon indicate diversity

**Table S4.** Numbers of links in the bacterial networks obtained for different rhizosphere soil and endophytic samples in Ejinur Salt Lake.

|  | GA | GB | GC | GD | RA | RB | RC | RD |
| --- | --- | --- | --- | --- | --- | --- | --- | --- |
| Total links | 365 | 402 | 403 | 412 | 497 | 448 | 616 | 401 |
| Positive links | 188(51.5%) | 193(48.0%) | 197(48.9%) | 217(52.7%) | 298(60.0%) | 274(61.2%) | 323(52.4%) | 194(48.4%) |
| Negative links | 177(48.5%) | 209(52.0%) | 206(51.1%) | 195(47.3%) | 208(40.0%) | 174(38.8%) | 293(47.6%) | 207(51.6%) |

GA-GD, rhizosphere soil samples; RA-RD, endophytic samples. The percentages in brackets represent the proportion of each number of links relative to the total links for each sample. *Salicornia europaea* (A), *Suaeda salsa* (B), *Phragmites communis* (C) and *Achnatherum splendens* (D).

**Table S5.** Numbers of links in the fungal networks obtained for different rhizosphere soil and endophytic samples in Ejinur Salt Lake.

|  | GA | GB | GC | GD | RA | RB | RC | RD |
| --- | --- | --- | --- | --- | --- | --- | --- | --- |
| Total links | 362 | 355 | 297 | 353 | 21 | 465 | 442 | 181 |
| Positive links | 285(78.7%) | 286(80.6%) | 195(65.7%) | 187(53.0%) | 18(85.7%) | 416(89.5%) | 432(97.7%) | 115(63.5%) |
| Negative links | 77(21.3%) | 69(19.4%) | 102(34.3%) | 166(47.0%) | 3(14.3%) | 49(10.5%) | 10(2.3%) | 66(36.5%) |

GA-GD, rhizosphere soil samples; RA-RD, endophytic samples. The percentages in brackets represent the proportion of each number of links relative to the total links for each sample. *Salicornia europaea* (A), *Suaeda salsa* (B), *Phragmites communis* (C) and *Achnatherum splendens* (D)

|  | Phylum | A (%) | B (%) | C (%) | D (%) |
| --- | --- | --- | --- | --- | --- |
| Bacterial rhizosphere | Proteobacteria | 36.03±2.55 b | 40.22±2.21 a | 31.55±0.12 c | 34.46±2.08 bc |
|  | Actinobacteria | 7.99±1.96 d | 16.92±0.72 c | 26.49±0.24 b | 35.35±1.93 a |
|  | Bacteroidetes | 25.95±3.44 a | 15.80±1.83 b | 10.86±0.56 c | 6.46±0.29 d |
|  | Firmicutes | 11.29±3.18 a | 7.55±1.21 b | 9.81±0.85 ab | 2.87±0.39 c |
|  | Chloroflexi | 5.23±0.98 a | 6.39±1.78 a | 7.08±0.11 a | 6.61±1.22 a |
|  | Gemmatimonadetes | 1.96±0.20 c | 2.36±0.42 b | 2.43±0.21 bc | 4.35±0.20 a |
|  | Patescibacteria | 1.65±0.14 c | 1.85±0.46 bc | 2.69±0.24 a | 2.23±0.08 ab |
|  | Acidobacteria | 0.75±0.07 b | 0.70±0.10 b | 2.59±0.48 a | 3.30±0.78 a |
|  | Others | 9.16±2.13 a | 7.94±2.27 a | 6.51±0.85 ab | 4.38±1.24 b |
| Bacterial endosphere | Proteobacteria | 64.56±4.21 A | 58.50±8.27 AB | 49.62±1.35 B | 52.10±7.00 B |
|  | Actinobacteria | 5.78±3.89 B | 32.01±4.63 A | 18.76±13.52 AB | 25.73±5.50 A |
|  | Bacteroidetes | 18.17±6.12 A | 1.16±0.51 B | 21.83±8.97 A | 15.24±2.24 A |
|  | Firmicutes | 5.74±7.03 A | 8.03±5.01 A | 6.81±5.89 A | 1.25±1.32 A |
|  | Planctomycetes | 1.57±0.58 A | 0.03±0.02 B | 0.84±0.77 AB | 1.08±0.29 A |
|  | Chloroflexi | 0.23±0.09 B | 0.05±0.05 B | 0.18±0.15 B | 2.25±0.30 A |
|  | Acidobacteria | 0.74±0.48 A | 0.03±0.05 B | 0.31±0.12 AB | 0.63±0.39 AB |
|  | Gemmatimonadetes | 0.19±0.12 B | 0.04±0.05 B | 0.49±0.61 AB | 0.91±0.34 A |
|  | Others | 3.01±1.25 A | 0.15±0.03 B | 1.16±0.46 B | 0.82±0.22 B |

**Table S6.** The main phylum of bacteria in rhizospheric samples and endophytic samples and their relative abundance.

Data are means ± standard deviation (n=3). Values in the same row followed by the same letter(s) are not significantly different at *P* < 0.05. Different uppercase (Endophytic samples) and lowercase (Rhizospheric samples) letters indicate significant differences among plant species. Letters indicate the plant species (*Salicornia europaea* (A), *Suaeda salsa* (B), *Phragmites communis* (C) and *Achnatherum splendens* (D))

**Table S7.** The main phylum of fungi in rhizospheric samples and endophytic samples and their relative abundance.

|  | Phylum | A | B | C | D |
| --- | --- | --- | --- | --- | --- |
| Fungal rhizosphere | Ascomycota | 94.80±1.61 a | 96.79±0.99 a | 94.30±1.69 a | 95.44±0.68 a |
|  | Basidiomycota | 3.38±1.59 ab | 2.11±0.40 b | 5.48±1.53 a | 3.03±0.08 b |
|  | unclassified_k_Fungi | 1.78±0.42 a | 1.07±0.60 ab | 0.21±0.16 ab | 1.11±0.53 b |
|  | Others | 0.04±0.00 b | 0.03±0.00 b | 0.01±0.01 b | 0.42±0.17 a |
| Fungal endosphere | Ascomycota | 99.98±0.00 A | 98.62±1.69 A | 72.65±47.19 A | 99.87±0.04 A |
|  | Basidiomycota | 0.00±0.00 A | 0.99±1.04 A | 27.32±47.16 A | 0.05±0.05 A |
|  | unclassified_k_Fungi | 0.02±0.01 A | 0.38±0.64 A | 0.01±0.02 A | 0.02±0.02 A |
|  | Others | 0.00±0.01 B | 0.01±0.01 B | 0.01±0.02 B | 0.06±0.03 A |

Data are means ± standard deviation (n=3). Values in the same row followed by the same letter(s) are not significantly different at *P* < 0.05. Different uppercase (Endophytic samples) and lowercase (Rhizospheric samples) letters indicate significant differences among plant species. Letters indicate the plant species (*Salicornia europaea* (A), *Suaeda salsa* (B), *Phragmites communis* (C) and *Achnatherum splendens* (D))

|  | Metabolic  Pathway | A (%) | B (%) | C (%) | D (%) |
| --- | --- | --- | --- | --- | --- |
| Bacterial  rhizosphere | Aerobic respiration I (cytochrome c) | 1.45±0.01 d | 1.49±0.01 c | 1.62±0.00 b | 1.69±0.01 a |
|  | Pyruvate fermentation to isobutanol (engineered) | 0.90±0.01 c | 0.91±0.01 c | 0.96±0.01 b | 1.00±0.01 a |
|  | L-isoleucine biosynthesis II | 0.91±0.01 c | 0.91±0.01 c | 0.95±0.01 b | 0.98±0.01 a |
|  | L-isoleucine biosynthesis I (from threonine) | 0.89±0.01 c | 0.88±0.01 c | 0.92±0.01 b | 0.95±0.01 a |
|  | L-valine biosynthesis | 0.89±0.01 c | 0.88±0.01 c | 0.92±0.01 b | 0.95±0.01 a |
|  | Superpathway of branched amino acid biosynthesis | 0.79±0.01 b | 0.79±0.01 b | 0.83±0.00 a | 0.84±0.01 a |
|  | Gondoate biosynthesis (anaerobic) | 0.80±0.02 a | 0.75±0.02 b | 0.70±0.00 c | 0.67±0.01 d |
|  | L-isoleucine biosynthesis III | 0.70±0.01 b | 0.70±0.01 b | 0.74±0.00 a | 0.73±0.01 a |
|  | Cis-vaccenate biosynthesis | 0.77±0.01 a | 0.72±0.01 b | 0.70±0.00 c | 0.68±0.01 c |
|  | CDP-diacylglycerol biosynthesis II | 0.70±0.00 b | 0.69±0.01 c | 0.71±0.00 a | 0.70±0.00 ab |
| Bacterial  endosphere | Aerobic respiration I (cytochrome c) | 1.47±0.08 bc | 1.39±0.03 c | 1.53±0.05 b | 1.81±0.07 a |
|  | Pyruvate fermentation to isobutanol (engineered) | 1.02±0.01 b | 1.21±0.04 a | 0.97±0.04 bc | 0.93±0.01 c |
|  | L-isoleucine biosynthesis II | 1.00±0.04 ab | 1.02±0.08 a | 0.95±0.07 ab | 0.91±0.01 b |
|  | L-isoleucine biosynthesis I (from threonine) | 0.99±0.04 ab | 1.04±0.10 a | 0.95±0.05 ab | 0.88±0.01 b |
|  | L-valine biosynthesis | 0.99±0.04 ab | 1.04±0.10 a | 0.95±0.05 ab | 0.88±0.01 b |
|  | Gondoate biosynthesis (anaerobic) | 0.93±0.07 a | 0.69±0.04 b | 0.87±0.09 a | 0.85±0.03 a |
|  | Superpathway of branched amino acid biosynthesis | 0.83±0.04 a | 0.83±0.05 a | 0.78±0.07 a | 0.79±0.01 a |
|  | Cis-vaccenate biosynthesis | 0.84±0.02 a | 0.66±0.03 b | 0.84±0.09 a | 0.81±0.02 a |
|  | Fatty acid elongation - saturated | 0.84±0.06 a | 0.67±0.04 b | 0.79±0.07 a | 0.81±0.01 a |
|  | L-isoleucine biosynthesis III | 0.72±0.03 a | 0.72±0.02 a | 0.66±0.06 a | 0.70±0.01 a |

**Table S8.** Average relative abundance of 10 Metabolic Pathway of bacteria in rhizospheric and endophytic samples.

Data are means ± standard deviation (n=3). Values in the same row followed by the same letter(s) are not significantly different at *P* < 0.05. Letters indicate the plant species (*Salicornia europaea* (A), *Suaeda salsa* (B), *Phragmites communis* (C) and *Achnatherum splendens* (D)).

**Table S9.** Average relative abundance of 10 Metabolic Pathway of fungi in rhizospheric and endophytic samples.

Data are means ± standard deviation (n=3). Values in the same row followed by the same letter(s) are not significantly different at *P* < 0.05. Letters indicate the plant species (*Salicornia europaea* (A), *Suaeda salsa* (B), *Phragmites communis* (C) and *Achnatherum splendens* (D)).

|  | Metabolic  Pathway | A (%) | B (%) | C (%) | D (%) |
| --- | --- | --- | --- | --- | --- |
| Fungal  rhizosphere | Aerobic respiration I (cytochrome c) | 6.33±0.05 a | 5.57±0.18 b | 6.34±0.02 a | 2.16±0.16 c |
|  | Aerobic respiration II (cytochrome c) (yeast) | 6.33±0.05 a | 5.57±0.18 b | 6.34±0.02 a | 2.16±0.16 c |
|  | Palmitate biosynthesis I (animals and fungi) | 1.96±0.03 c | 2.42±0.15 b | 2.03±0.03 c | 11.03±0.34a |
|  | Fatty acid and beta-oxidation (peroxisome, yeast) | 3.16±0.07 b | 3.36±0.08 a | 2.44±0.02 c | 3.40±0.04 a |
|  | Glyoxylate cycle | 3.35±0.04 a | 3.31±0.00 a | 3.17±0.02 b | 2.38±0.01 c |
|  | Pentose phosphate pathway (non-oxidative branch) | 2.68±0.03 b | 2.94±0.03 a | 2.71±0.04 b | 2.03±0.01 c |
|  | D-myo-inositol (1,4,5)-trisphosphate biosynthesis | 2.40±0.01 b | 2.21±0.02 c | 2.62±0.05 a | 1.91±0.02 d |
|  | GDP-mannose biosynthesis | 2.47±0.01 b | 1.88±0.03 c | 2.75±0.03 a | 1.66±0.03 d |
|  | Methyl ketone biosynthesis | 2.03±0.11 c | 1.93±0.03 c | 2.19±0.03 b | 2.36±0.00 a |
|  | Guanosine nucleotides degradation II | 2.59±0.02 b | 2.28±0.03 c | 2.85±0.03 a | 1.04±0.08 d |
| Fungal  endosphere | Aerobic respiration I (cytochrome c) | 6.45±0.09 ab | 6.04±0.20 b | 6.84±0.43 a | 5.01±0.16 c |
|  | Aerobic respiration II (cytochrome c) (yeast) | 6.45±0.09 ab | 6.04±0.20 b | 6.84±0.43 a | 5.01±0.16 c |
|  | Glyoxylate cycle | 3.11±0.06 b | 3.35±0.12 a | 3.24±0.07 ab | 3.23±0.10 ab |
|  | Fatty acid and beta-oxidation (peroxisome, yeast) | 2.44±0.19 a | 3.24±0.33 a | 2.93±1.02 a | 2.29±0.05 a |
|  | D-myo-inositol (1,4,5)-trisphosphate biosynthesis | 2.38±0.07 b | 2.25±0.13 b | 2.85±0.17 a | 3.08±0.15 a |
|  | Guanosine nucleotides degradation II | 2.78±0.05 a | 2.25±0.27 b | 2.73±0.40 ab | 2.71±0.19 ab |
|  | Pentose phosphate pathway (non-oxidative branch) | 2.83±0.05 a | 2.95±0.21 a | 2.40±0.19 b | 1.74±0.04 c |
|  | TCA cycle II (plants and fungi) | 2.33±0.05 b | 2.16±0.04 c | 2.45±0.06 a | 2.33±0.06 b |
|  | GDP-mannose biosynthesis | 2.96±0.18 a | 1.92±0.09 c | 2.39±0.40 b | 1.87±0.02 c |
|  | Palmitate biosynthesis I (animals and fungi) | 2.14±0.05 ab | 2.13±0.08 ab | 1.56±1.08 b | 3.00±0.26 a |


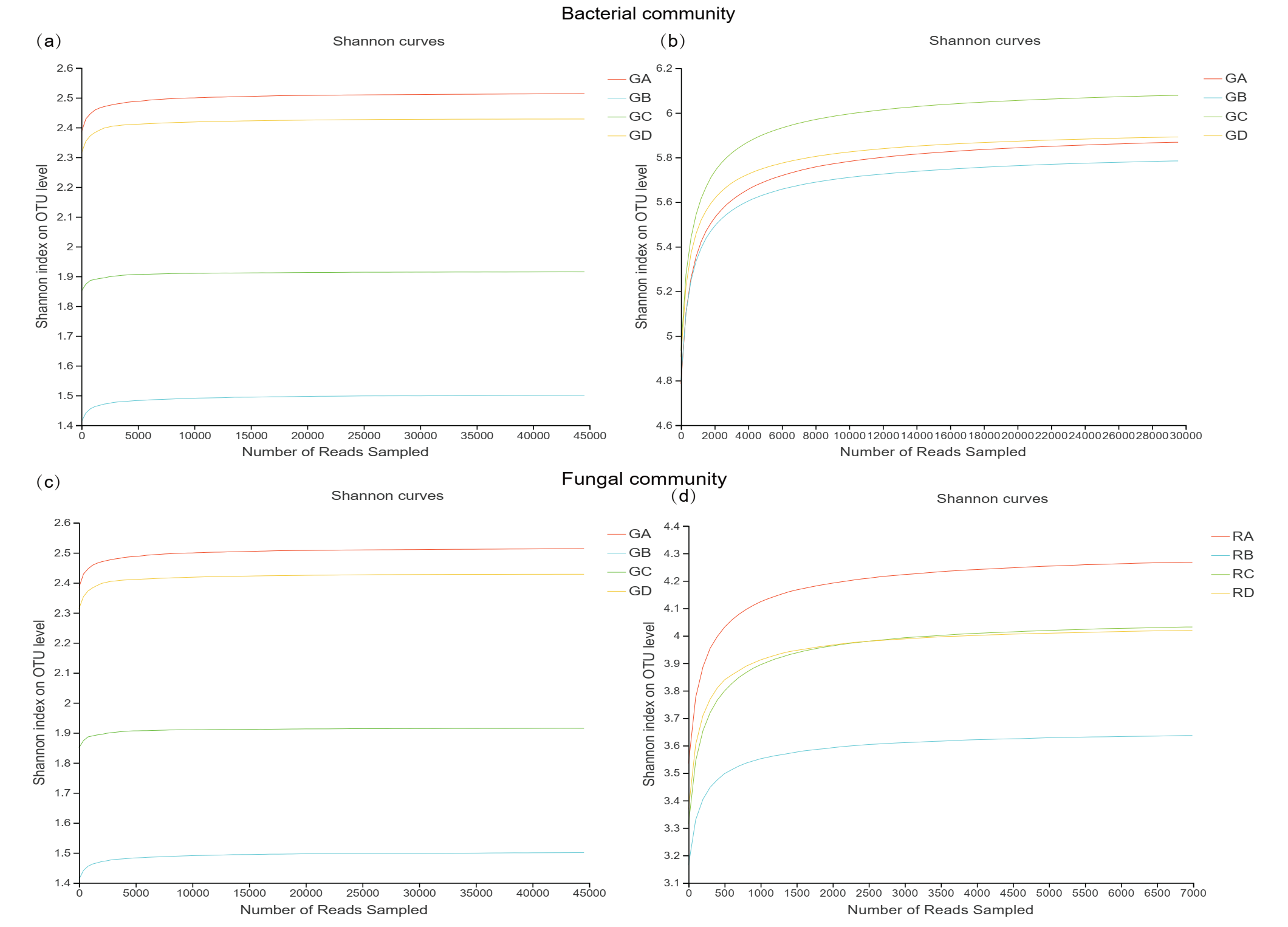


**Fig. S1.** Dilution curve for observed rhizosphere soil and endophytic bacteria and fungi OTUs reach a flat level. a-b (Bacterial community), c-d (Fungal community). GA-GD, rhizosphere soil samples; RA-RD, endophytic samples. *Salicornia europaea* (A), *Suaeda salsa* (B), *Phragmites communis* (C) and *Achnatherum splendens* (D).


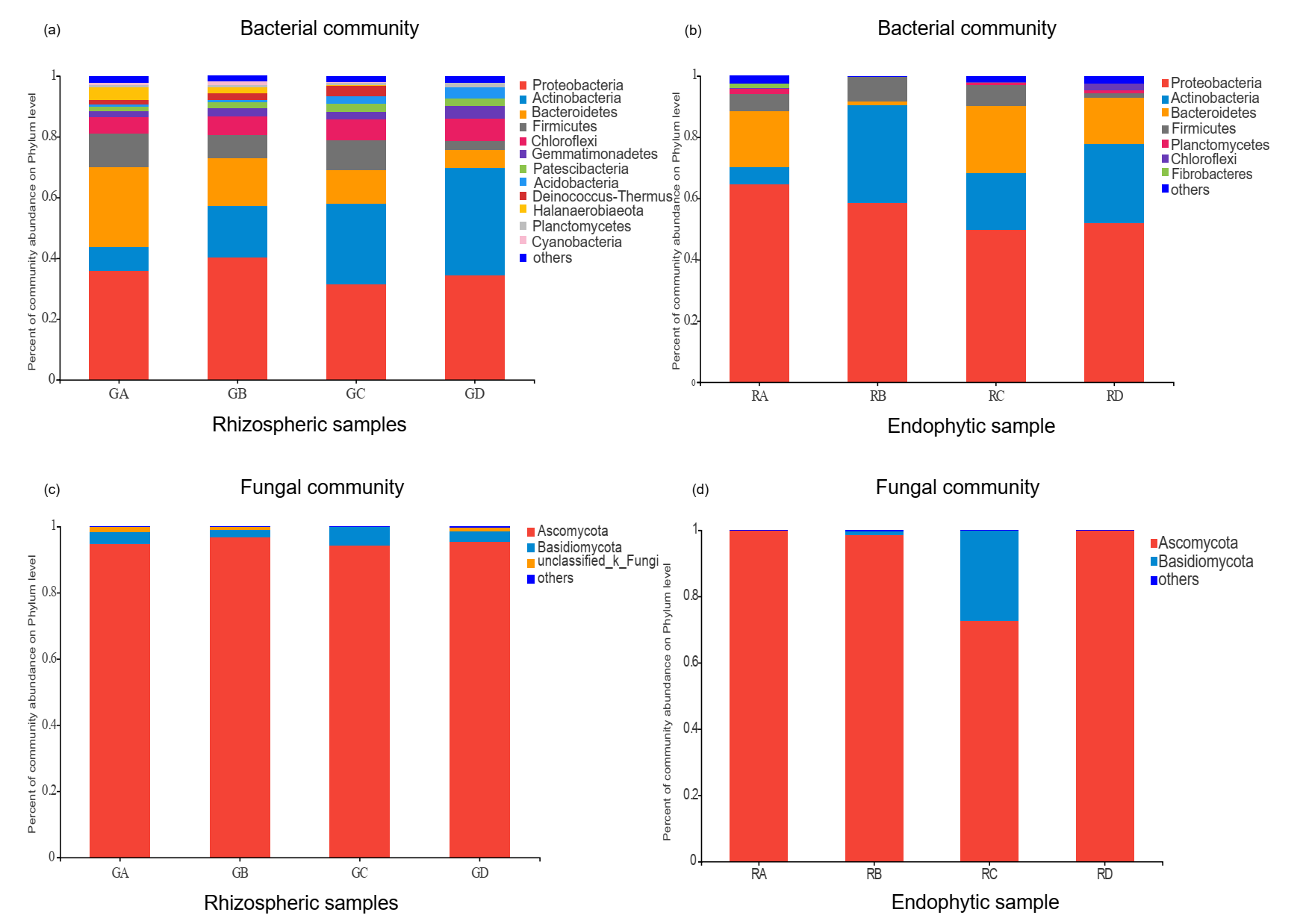


**Fig. S2.** Effects of rhizosphere soil and plant endophytic samples on the community composition at the phylum level of Bacteria and Fungi in the Ejinur Salt Lake. Phyla with relative abundance less than 1% were defined as others. (a) bacterial rhizospheric samples, (b) bacterial endophytic samples (c) fungal rhizospheric samples (d) fungal endophytic samples. *Salicornia europaea* (A), *Suaeda salsa* (B), *Phragmites communis* (C) and *Achnatherum splendens* (D)


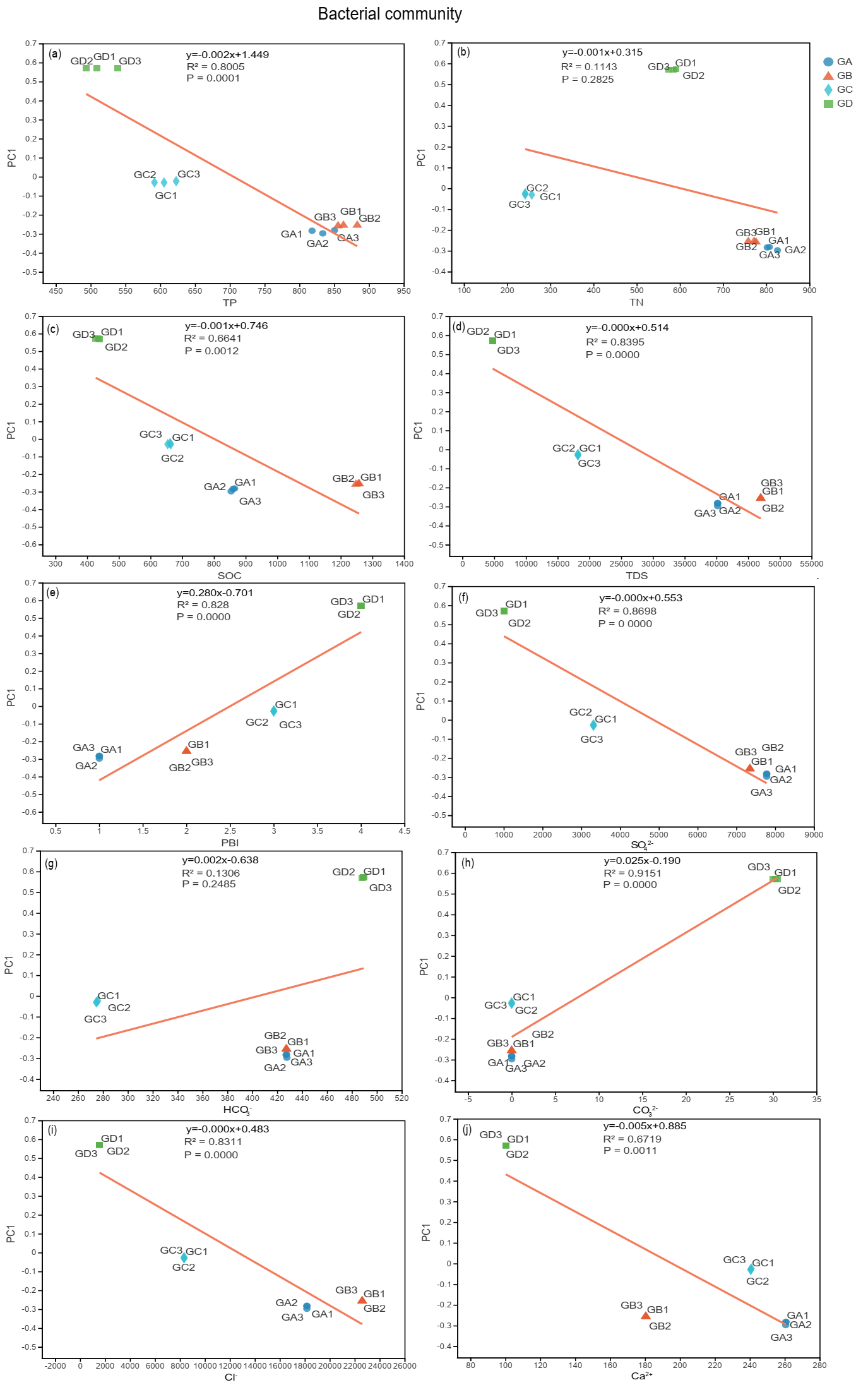


**Fig. S3.** Linear correlation between plant species (PBI) and soil environmental factors and *β*-diversity (PCoA based on Bray-Curtis) of bacterial rhizospheric samples. *Salicornia europaea* rhizosphere soils (GA), *Suaeda salsa* rhizosphere soils (GB), *Phragmites communis* rhizosphere soils (GC) and *Achnatherum splendens* rhizosphere soils (GD).


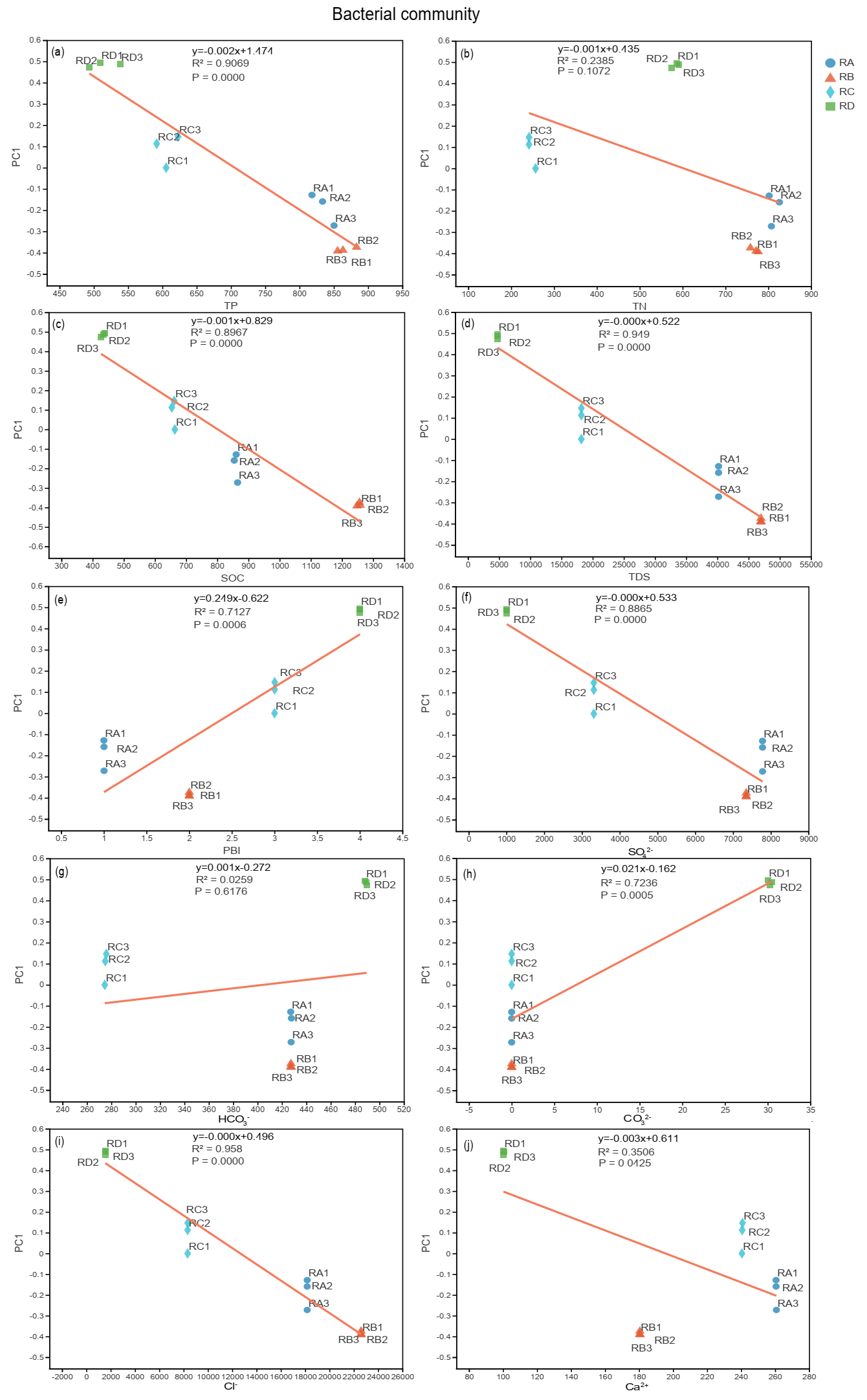


**Fig. S4.** Linear correlation between plant species (PBI) and soil environmental factors and *β*-diversity (PCoA based on Bray-Curtis) of bacterial endophytic samples. *Salicornia europaea* endophytic samples (RA), *Suaeda salsa* endophytic samples (RB), *Phragmites communis* endophytic samples (RC) and *Achnatherum splendens* endophytic samples (RD).


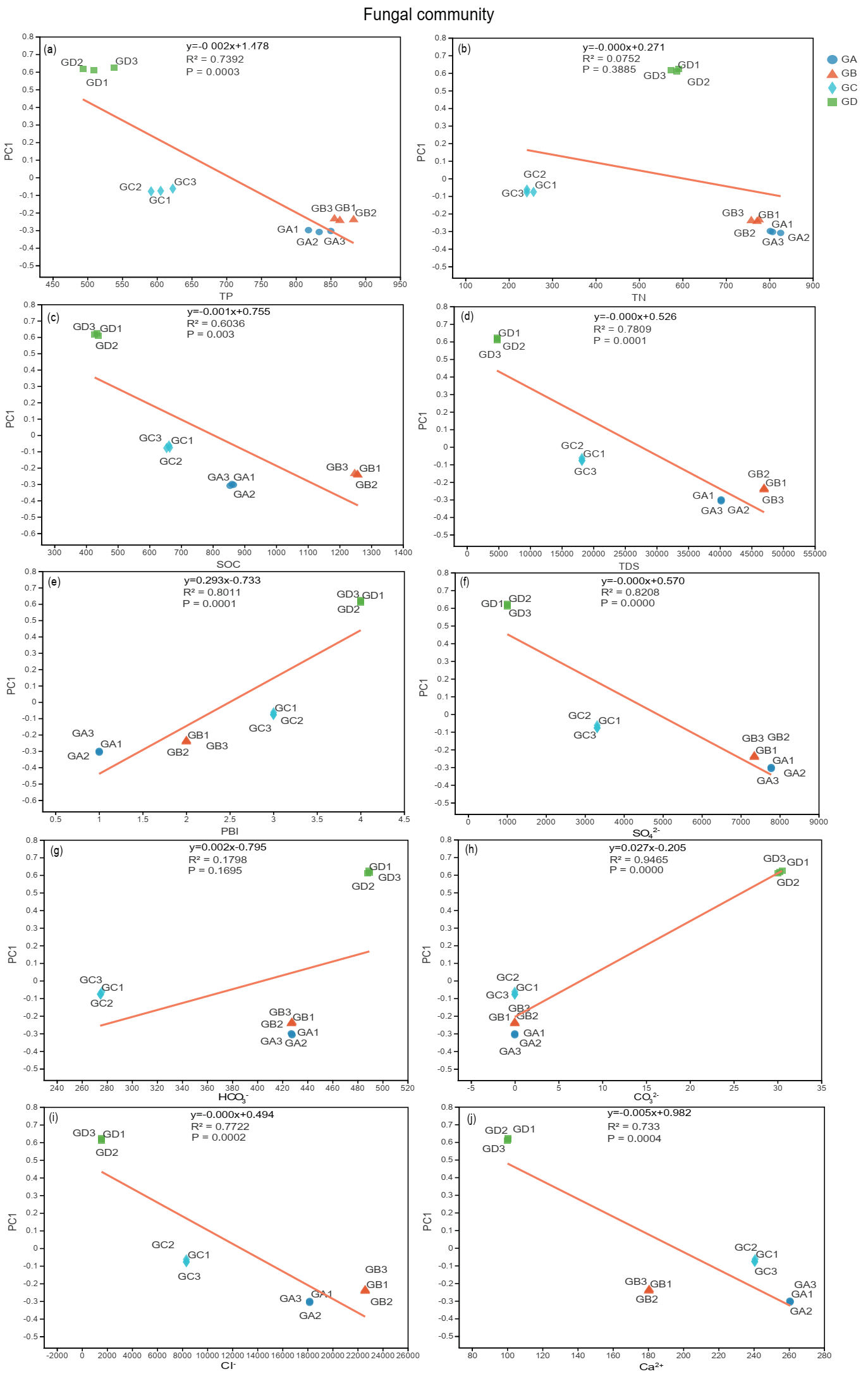


**Fig. S5.** Linear correlation between plant species (PBI) and soil environmental factors and *β*-diversity (PCoA based on Bray-Curtis) of fungal rhizospheric samples. *Salicornia europaea* rhizosphere soils (GA), *Suaeda salsa* rhizosphere soils (GB), *Phragmites communis* rhizosphere soils (GC) and *Achnatherum splendens* rhizosphere soils (GD).


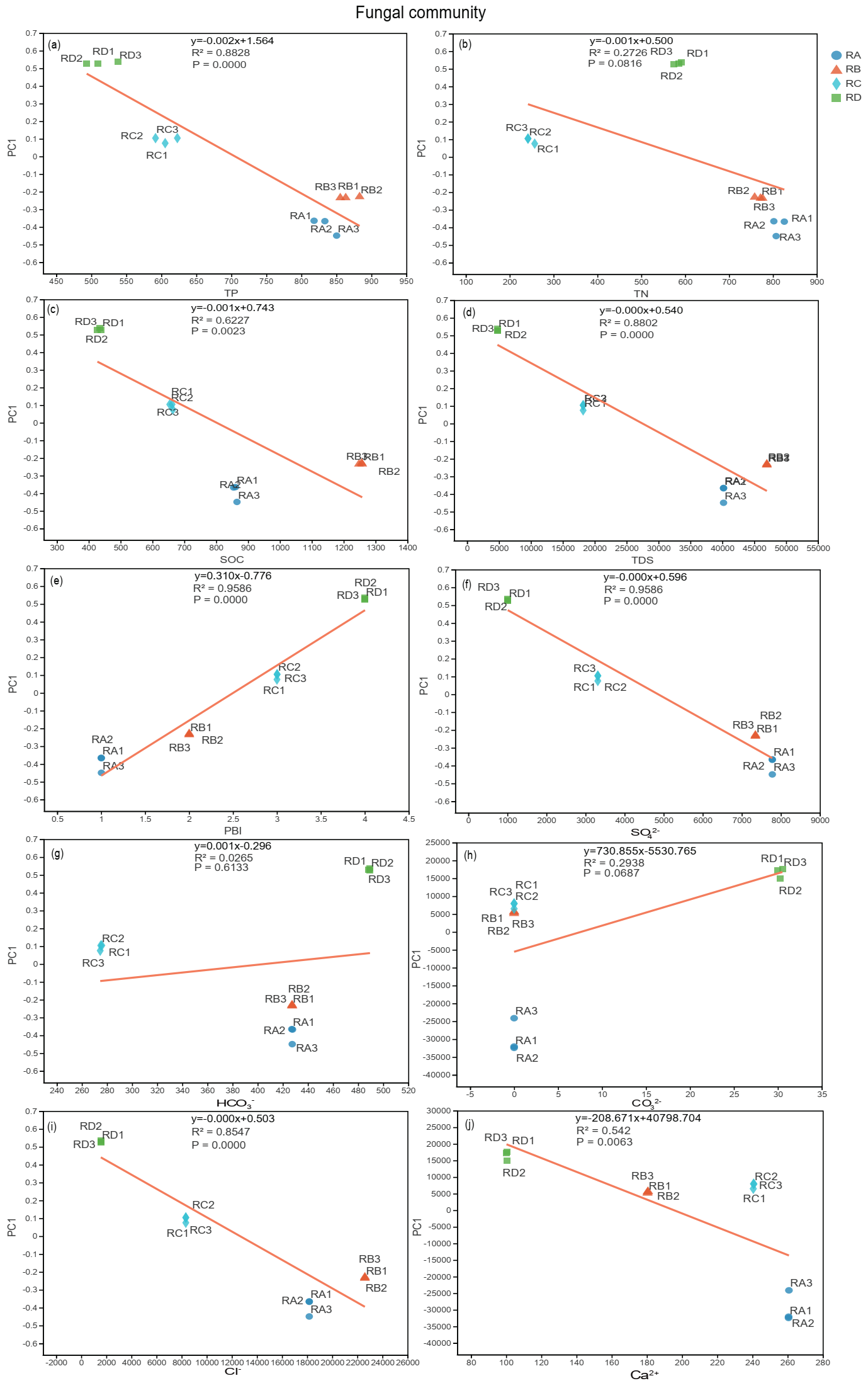
 **Fig. S6.** Linear correlation between plant species (PBI) and soil environmental factors and *β*-diversity (PCoA based on Bray-Curtis) of fungal endophytic samples. *Salicornia europaea* endophytic samples (RA), *Suaeda salsa* endophytic samples (RB), *Phragmites communis* endophytic samples (RC) and *Achnatherum splendens* endophytic samples (RD).


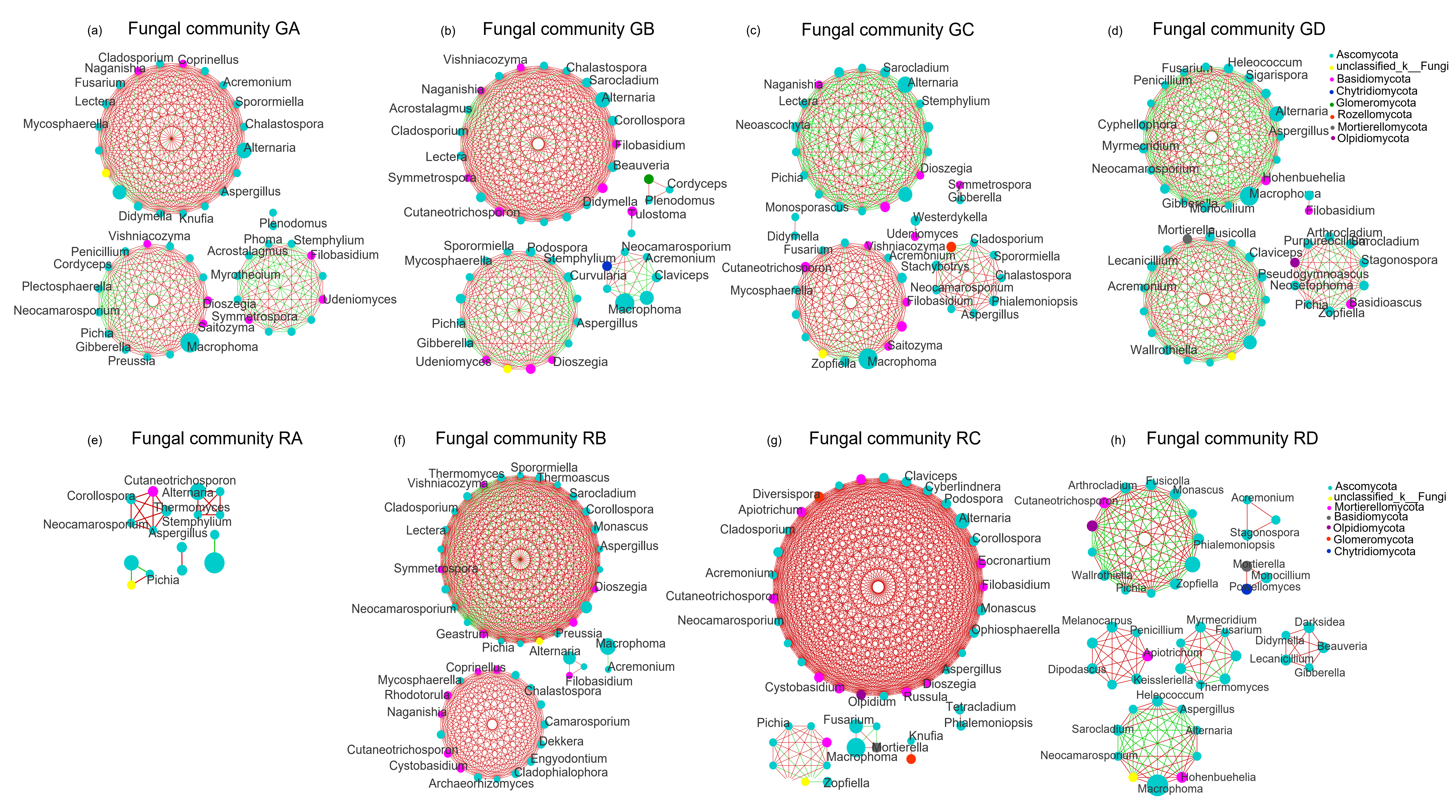


**Fig. S7.** An overview of the fungal network interactions in Ejinur Salt Lake different rhizosphere soil and endophytic samples. The connections in the network represent a strong (|*r*| > 0.6) and significant (*P* < 0.01) correlations Colors of nodes represent different major phyla, the node representing genus is written inside. A green link indicates a negative interaction, whereas a red link implies a positive interaction between two individual nodes. a-d: *Salicornia europaea* rhizosphere soil (GA), *Suaeda salsa* rhizosphere soil (GB), *Phragmites communis* rhizosphere soil (GC) and *Achnatherum splendens* rhizosphere soil (GD). e-h: *Salicornia europaea* endophytic samples (RA), *Suaeda salsa* endophytic samples (RB), *Phragmites communis* endophytic samples (RC) and *Achnatherum splendens* endophytic samples (RD).
